# MSX1 variant causes nonsyndromic tooth agenesis in a Japanese patient

**DOI:** 10.1038/s41439-026-00343-5

**Published:** 2026-04-02

**Authors:** Yasuto Sano, Michiyo Ando, Reiko Tokuyama-Toda, Akiko Ota, Junichiro Machida, Kazuhito Satomura, Mitsuo Goto, Yoshihito Tokita

**Affiliations:** 1 Department of Oral and Maxillofacial Surgery, School of Dentistry, Aichi Gakuin University, Nagoya, Japan; 2 Department of Disease Model, Institute for Developmental Research, Aichi Developmental Disability Center, Kasugai, Japan; 3 Department of Oral Medicine and Stomatology, School of Dental Medicine, Tsurumi University, Yokohama, Japan; 4 Department of Oncology, Toyota Memorial Hospital, Toyota, Japan; 5 Department of Oral and Maxillofacial Surgery, Toyota Memorial Hospital, Toyota, Japan

**Keywords:** Diseases, Pathogenesis

## Abstract

MSX1 variants are associated with autosomal dominant craniofacial developmental anomalies, including congenital tooth agenesis. Here, whole-exome sequencing in a Japanese patient with congenital tooth agenesis identified a novel de novo heterozygous frameshift variant in MSX1 (NM_002448.3:c.299delC). The variant is predicted to generate a truncated protein, p.(Pro100Argfs*60), that lacks the C-terminal region of MSX1 and is expected to result in loss of function. The patient presented with congenital tooth agenesis, including canine agenesis. Taken together with the genetic findings, this case supports the involvement of MSX1 in congenital tooth agenesis and broadens the reported phenotypic presentation of MSX1-related tooth agenesis. This report adds to the spectrum of disease-associated MSX1 variants and supports the utility of genomic testing for molecular diagnosis in rare dental developmental anomalies.

## Data report

Congenital tooth agenesis is a common craniofacial anomaly and is classified into two subtypes based on the number of missing permanent teeth, excluding third molars. Hypodontia refers to the absence of one to five permanent teeth, whereas oligodontia indicates the absence of six or more permanent teeth (OMIM #106600, #604625, #150400, #167416 and #616724)^1^. More than 350 genes are involved in tooth development, and their functions must be precisely regulated in time, space and dosage for normal odontogenesis^2–4^. Previous studies have reported that human congenital tooth agenesis arises from pathogenic variants in *WNT10A*, *LRP6* and *AXIN2*, which participate in the Wnt–β-catenin signaling pathway, as well as in *MSX1*, *PAX9*, *EDA*, *EDAR* and *EDARADD*^5–10^. During early human development, these genes play crucial roles in tooth formation and the development of ectodermal tissue; consequently, variants in these genes can result in both nonsyndromic and syndromic forms of tooth dysplasia. Although the loss of a small number of teeth may have a limited clinical impact, more extensive agenesis can cause functional and aesthetic impairment, as well as maxillofacial morphological abnormalities, leading to a substantial psychological and physical burden for affected individuals^1,11^.

MSX1, a transcriptional regulator, is essential for organogenesis by controlling the expression of downstream genes^12^. In the MSX1 protein, tooth-number abnormalities have been associated with variants that affect multiple functional domains. The homeodomain adopts a three-helix structure, in which the second helix contributes to structural stability, whereas the third helix mediates DNA binding^13,14^. The wild-type MSX1 protein localizes to the nucleus, and we previously identified two nuclear localization signals within the homeodomain^15^, highlighting its role in transcriptional regulation. Notably, proper MSX1 function does not depend solely on the homeodomain. Pathogenic variants affecting the C-terminal MH6 domain (also referred to as the PIAS domain) have been reported to cause tooth agenesis even without directly disrupting the homeodomain^16^. The MH6 domain is thought to contribute to transcriptional regulation and DNA-binding specificity; thus, its functional impairment may also disturb odontogenesis.

**Fig. 1:**
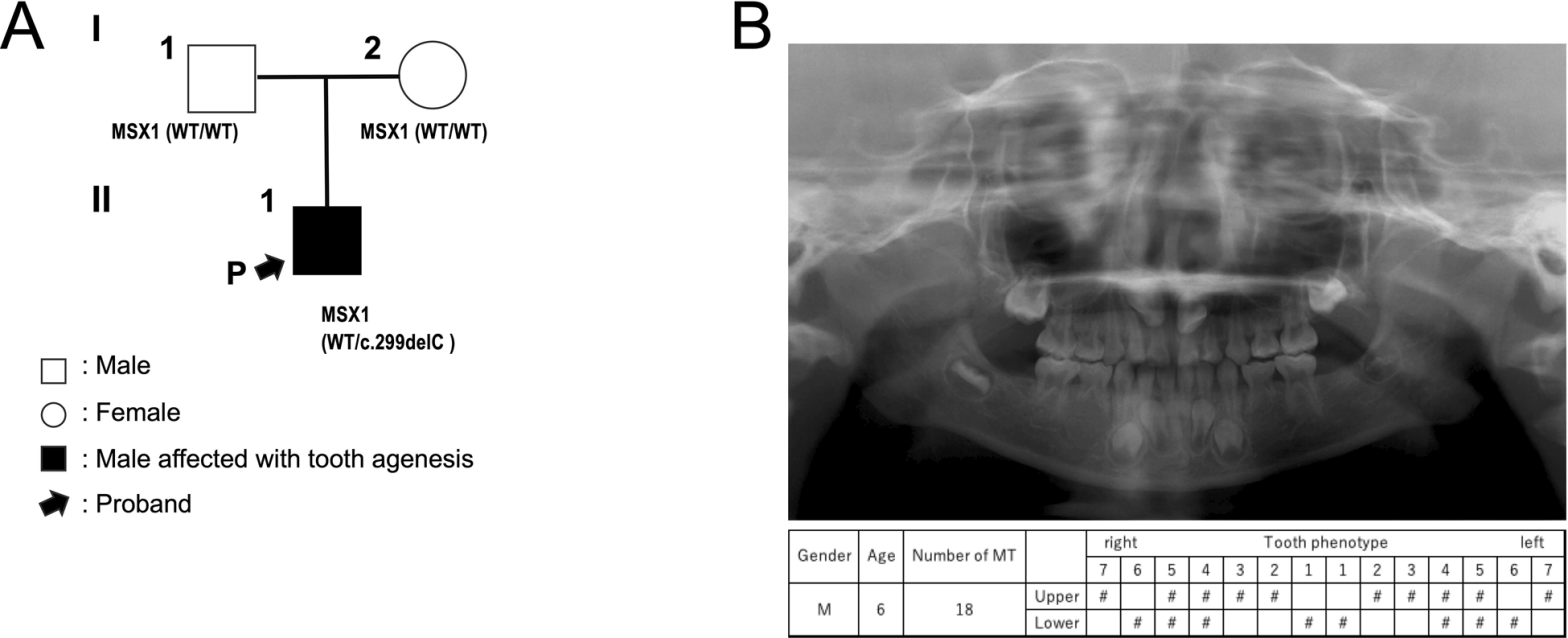
Pedigree and dental findings of the patient. **A** Pedigree of the family. The arrow indicates the proband. **B** Top: panoramic radiograph of the proband. Bottom: schematic representation of the patient’s permanent dentition. The symbol ‘#’ indicates congenitally missing teeth.

The patient was a 6-year-old boy with congenital tooth agenesis, born to nonconsanguineous Japanese parents. Neither he nor his family members had a history of craniofacial anomalies, including cleft lip and/or palate, or congenital disorders of ectodermal origin (Fig. 1A, B).

The study was approved by the institutional review boards of Aichi Gakuin University, Tsurumi University, and the Institute for Developmental Research, Aichi Developmental Disability Center, and written informed consent was obtained from the patient and his parents. Using the Oragene DISCOVER kit (OGR-600; DNA Genotek), 2 ml of saliva was noninvasively collected from the patient and his parents, and genomic DNA was extracted according to the manufacturer’s protocol. A DNA library was prepared using the SureSelect Human All Exon Kit (Agilent Technologies), and whole-exome sequencing was performed on the proband and his parents using an Illumina NovaSeq 6000 system (Illumina) to generate 150-bp paired-end reads.

In addition, MSX1 was analyzed by Sanger sequencing using specific primer sets (forward, 5′-AAGCCCAAAGTGTCCCCTTC-3′; reverse, 5′-GTCTGGAACCTTCTTCCTGGG-3′) with TaKaRa LA Taq DNA polymerase (Takara Bio) in the proband and both parents.

Using a trio-based de novo model, we screened the variant call file for high-impact variants—those predicted to severely affect protein structure or function—in candidate genes for tooth agenesis. We identified a single predicted loss-of-function variant: a heterozygous frameshift variant in MSX1 (NM_002448.3:c.299delC). Additional de novo variants were detected in untranslated regions or introns of the following genes: *ANTXR1*, *CDH1*, *COL1A1*, *CREBBP*, *DSP*, *EDA*, *EDAR*, *ERCC2*, *FOXC1*, *GTF2E2*, *KDM6A* and *P4HA3*. The MSX1 variant was not listed in population databases, including the Genome Aggregation Database (gnomAD), Exome Aggregation Consortium (ExAC), dbSNP, the 1000 Genomes Project or the NHLBI Exome Variant Server. According to the variant-classification guidelines of the American College of Medical Genetics and Genomics, the Association for Molecular Pathology and the College of American Pathologists^17^, this variant meets criteria PVS1 and PS2 and is therefore classified as pathogenic.

**Fig. 2:**
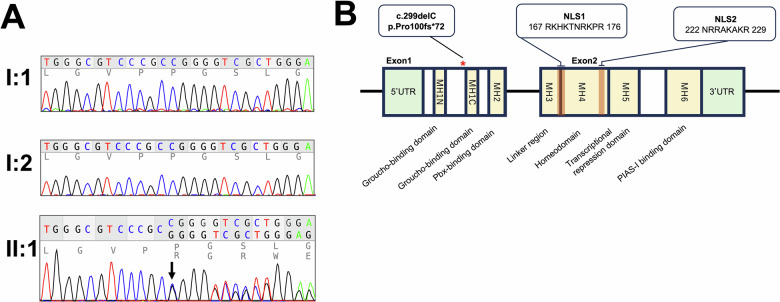
The MSX1 C-terminal deletion variant p.(Pro100ArgfsTer60). **A** Electropherogram from Sanger sequencing of the MSX1 gene. The arrow indicates the deletion site (c.299delC). **B** Schematic representation of the human MSX1 gene. The nucleotide deletion in the exon 1 (asterisk) introduces a frameshift mutation, leading to C-terminal truncation of MSX1 and predicted disruption of multiple functional domains, including the homeodomain.

The novel nucleotide deletion c.299delC identified in this study is located in exon 1. This deletion causes a frameshift that generates a truncated protein with an aberrant 60-amino-acid C-terminal tail, p.(Pro100Argfs*60). The C terminus of the MSX1 structure plays a pivotal role, as the homeodomain and the C-terminal MH6 (PIAS) domain potentially compromise multiple functional regions implicated in transcriptional regulation, including DNA binding and nuclear localization^15–18^. MSX1 haploinsufficiency has been shown to decrease BMP4 and LEF1 signaling in the interdental lobe during tooth development^19^. In other words, the c.299delC variant is expected to fail to transmit sufficient signaling for normal tooth germ development, and MSX1 haploinsufficiency is likely to underlie tooth agenesis in the present case (Fig. 2).

In addition to this mechanistic interpretation, the present case highlights a relatively uncommon phenotypic feature of tooth agenesis, namely canine agenesis. In patients with variants in major causative genes such as *WNT10A* and *LRP6*, premolars and molars are more frequently affected, whereas canine agenesis is less common. Nevertheless, canine agenesis has been reported sporadically. For example, in a recent clinical and genetic analysis of *MSX1*-associated nonsyndromic tooth agenesis, Ding et al.^20^ documented maxillary right canine agenesis in one affected individual^20^. This observation further supports the phenotypic spectrum of MSX1-related tooth agenesis, which includes canine involvement, albeit at low frequency.

It remains unclear, however, how the precise number of teeth is determined. In addition to effects on protein structure, it will be essential to investigate other consequences of nucleotide substitutions on tooth phenotypes, such as the creation of unexpected microRNA target sites or alterations in the transcriptional activity of promoter regions.

## HGV Database

The relevant data from this Data Report are hosted at the Human Genome Variation Database at 10.6084/m9.figshare.hgv.3634.
